# Cardio-Pulmonary Histopathology with Clinical Correlations of Deceased Patients with COVID-19: A Case Series in Tehran, Iran

**DOI:** 10.34172/aim.2023.39

**Published:** 2023-05-01

**Authors:** Saeed Soleiman-Meigooni, Ramin Yaghmayee, Shadi Mohammadi, Mousa Ahmadi, Mehdi Sakhabakhsh, Ramin Hamidi-Farahani, Ebrahim Hazrati, Seyed Mohammad Jazayeri, Mahtab Fotoohi, Akram Motemaveleh, Vahid Doulatabadi-Farahani, Farhad Shahmohamadi, Mohammad Hassan Kazemi-Galougahi, Ali Asgari, Mohammad Aminianfar, Mohammad Darvishi, Mojgan Mohajeri-Iravani, Omid Gholizadeh

**Affiliations:** ^1^Infectious Diseases Research Center, Aja University of Medical Sciences, Tehran, Iran; ^2^Department of Pathology, Khanevadeh University Hospital, Aja University of Medical Sciences, Tehran, Iran; ^3^Department of Obstetrics and Gynecology, Khanevadeh University Hospital, Aja University of Medical Sciences, Tehran, Iran; ^4^Department of Infectious Diseases, Faculty of Medicine, Aja University of Medical Sciences, Tehran, Iran; ^5^Department of Neurology, Faculty of Medicine, Aja University of Medical Sciences, Tehran, Iran; ^6^Department of Anesthesiology, Faculty of Medicine, Aja University of Medical Sciences, Tehran, Iran; ^7^Research Center for Clinical Virology, Tehran University of Medical Sciences, Tehran, Iran; ^8^Department of Pulmonology, Khanevadeh University Hospital, Aja University of Medical Sciences, Tehran, Iran; ^9^Department of Cardiology, Khanevadeh University Hospital, Aja University of Medical Sciences, Tehran, Iran; ^10^Department of Forensic Medicine, Khanevadeh University Hospital, Aja University of Medical Sciences, Tehran, Iran; ^11^Department of Epidemiology, Faculty of Medicine, Aja University of Medical Sciences, Tehran, Iran; ^12^Department of Anesthesiology, Faculty of Paramedical Sciences, Aja University of Medical Sciences, Tehran, Iran; ^13^Research Center for Clinical Virology, Tehran University of Medical Sciences, Tehran, Iran

**Keywords:** Cardiac, Pathology, Pulmonary, SARS-CoV-2

## Abstract

**Background:**

SARS-CoV-2 may affect vital organs. The present study investigated the histopathology of pulmonary and cardiac tissues with clinical correlation in deceased patients with COVID-19.

**Methods:**

We obtained pulmonary and cardiac tissues from 30 deceased patients with COVID-19 in Tehran, Iran, from January to May 2021. Sampling was performed through a percutaneous needle biopsy. After slide preparation, two expert pathologists studied them. We assessed the correlation between clinical and pathological data by Fisher’s exact test.

**Results:**

The mean age of the patients was 73.8±13.4 years, and the male-to-female ratio was 23/7. The most common underlying disease was hypertension (HTN) in 25 patients (83%). Fifty-five tissue samples were achieved, including 28 pulmonary and 27 cardiac samples. Our results showed that all patients (100%) developed diffuse alveolar damage (DAD), and 26 (93%) developed hyaline membrane formation. The most common phase of DAD was the exudative-proliferative phase in 16 (57.1%). Three cardiac samples (11%) revealed myocarditis, and seven (26%) showed cardiomyocyte hypertrophy. In univariate analysis using Fischer’s exact test, myocarditis had significant relationships with C-reactive protein (CRP) levels higher than 80 mg/dL (*P*=0.008) and elevated cardiac troponin levels higher than two-fold (*P*=0.01).

**Conclusion:**

COVID-19 can affect the major vital organs. However, only myocarditis had a significant relationship with the circulating levels of inflammatory factors.

## Introduction

 SARS-CoV-2 enters the respiratory epithelial cells via angiotensin-converting enzyme II (ACE-II) receptors.^1^ Several chronic diseases such as diabetes mellitus (DM), hypertension (HTN), chronic lung disease (CLD), chronic renal failure (CRF), and ischemic heart disease (IHD) lead to increased expression of ACE-II receptors, higher viral binding, and a more severe immune response. This phenomenon explains the severe COVID-19 in the elderly with underlying diseases.^2,3^ The primary organ target of this virus is the lungs. In severe disease, respiratory distress and diffuse alveolar damage (DAD) constitute the leading cause of death.^4^ COVID-19 can affect other vital organs and cause fatal inflammation. Numerous cases of myocarditis and encephalitis have been reported in patients with COVID-19.^5-7^ Nevertheless, only a few studies performed multi-organ biopsies on these patients. We aimed to assess the clinical and histopathological features of pulmonary and cardiac tissues in deceased patients with COVID-19.

## Materials and Methods

###  Study Design

 We performed this case series on deceased patients with a polymerase chain reaction-confirmed COVID-19 in Khanevadeh Academic Hospital in Tehran, Iran, from January to May 2021.We selected our subjects by conventional sampling.

###  Participants and Sampling Methods

 We only selected deceased patients whose first-degree relatives gave written informed consent for sampling in that period. The bodies underwent minimal invasive percutaneous needle biopsy 6‒24 hours following death. An expert with a universal gun, two coats of gloves, a five-layered mask, and a face shield took the samples in a dissection room with negative pressure ventilation.^8^ A semi-automatic biopsy needle was used for cardiac and pulmonary sampling (Supplementary file 1, Figure S1). The pulmonary samples were taken from the areas with the most severe involvement on the computed tomography scan, often through the seventh or eighth intercostal space. The cardiac samples were taken through the fourth or fifth left anterior intercostal space.

###  Tissue Preparation

 The tissues were prepared by the formalin-fixed paraffin-embedded (FFPE) method and were cut into 10-μM sections using a microtome device. They were fixed on a slide and stained using hematoxylin and eosin dyes. Two expert pathologists studied each slide. The physicians and pathologists reviewed the histopathological findings and clinical data in three joint sessions.

## Results

###  Clinical and Laboratory Findings

 We included 30 deceased patients, including 23 men and 7 women. The mean age of the patients was 73.8 ± 13.4 years, and the mean body mass index (BMI) was 27.3 ± 3.1 kg/m^2^. The most common underlying diseases was HTN in 25 (83.3%), IHD in 21 (70%), DM in 13 (43.3%), CRF in 10 (33.3%), and CLD in 2 (6.7%). The most common symptoms of the patients were dyspnea in 27 (90%), cough in 22 (73.3%), malaise in 20 (66.7%), fever in 14 (46.7%), and decreased level of consciousness in 12 (40%). The time between the onset of symptoms and hospital admission was 6.2 ± 2.4 days, while the time between the onset of symptoms and death was 16.4 ± 9.4 days. All patients received supportive medication comprising anticoagulation, antiacid, broad-spectrum antibiotics, and bronchodilators during their ICU admission. Also, 27 patients (90%) received dexamethasone, and 21 (70%) received remdesivir. The extension of pulmonary ground glass opacity (GGO) on CT scans was less than 40% in 11 patients (36.7%), between 40 and 60% in 8 patients (26.6%), and over 60% in 11 patients (36.7%). Three patients (10%) developed vascular complications, consisting of deep vein thrombosis in one and ecchymosis in two. The most common clinical causes of death included respiratory distress due to viral pneumonia in 13 (43.3%), sepsis and multi-organ failure in 12 (40%), pulmonary embolism in 4 (13.3%), and myocarditis in one (3.3%). Table 1 summarizes the demographic characteristics, clinical and laboratory findings, and probable clinical causes of death in our patients.

**Table 1:** Main Clinical Data of the Study Population

**Case**	**Age**	**Gender**	**BMI** **(kg/m**^2^ **)**	**PMH**	**Main Symptoms**	**First SpO2**	**GGO on Lung CT**	**Days to Admission**	**Days to Death**	**WBC** **(×10**^3^ **U/L)**	**Hb** **(g/dL)**	**PLT** **(×10**^3^ **U/L)**	**CRP** **(mg/L)**	**D-Dimer** **(<200 ng/mL)**	**LDH** **(<450 U/L)**	**Trop** **(<34 ng/L)**	**Clinical Cause of Death**
1	77	M	27	HTN, IHD	Cough, dyspnea, malaise, LOC	85%	50-60%	5	10	9.5	11.9	90	23	210	340	12	ARDS
2	93	M	24	-	Fever, cough, dyspnea, malaise	80%	60-70%	9	12	17.7	13.8	162	68	560	693	15	ARDS
3	90	M	26	HTN, IHD, CRF	Cough, dyspnea, malaise, LOC	78%	60-70%	7	13	9.6	9.6	237	58	360	881	38	ARDS
4	83	M	25	HTN, IHD	Fever, cough, dyspnea, malaise	85%	70-80%	10	26	7.7	11	187	48	457	567	411	Sepsis
5	56	F	30	HTN	Dyspnea, malaise	78%	50-60%	8	16	2	12.4	110	5	8000	38	27	PTE
6	76	M	26	HTN, IHD, DM	Dyspnea, malaise, LOC	88%	40-50%	7	24	13	11	182	54	360	320	56	Sepsis
7	57	M	31	HTN, DM	Fever, cough, dyspnea, malaise	90%	70-80%	7	57	4.3	14	186	38	120	441	34	Sepsis
8	78	F	24	HTN, CRF	Cough, dyspnea, malaise	86%	30-40%	2	7	9.7	14.9	143	92	7480	765	785	ARDS
9	79	F	29	HTN, IHD, DM	Fever, dyspnea, LOC	92%	30-40%	7	12	8	12.1	317	2	230	310	14	ARDS
10	86	M	26	HTN, IHD, DM	Fever, LOC	88%	30-40%	6	14	2.4	12.4	83	58	880	550	11	PTE
11	79	F	28	HTN, IHD	Fever, cough, dyspnea, malaise, LOC	91%	20-30%	5	26	5.9	13	130	11	380	281	31	Sepsis
12	82	M	24	HTN, IHD	Fever, LOC	95%	10-20%	6	13	14.6	15	75	28	178	234	22	Sepsis
13	70	F	27	HTN	Fever, cough, dyspnea, malaise	75%	70-80%	9	17	3.7	14.4	136	65	1200	753	47	PTE
14	35	M	35	HTN, IHD, DM	Fever, cough, dyspnea, malaise	75%	70-80%	3	8	19.1	11.4	321	64	269	844	40	ARDS
15	64	M	31	DM	Cough, dyspnea, malaise	84%	70-80%	7	21	5.9	13.6	173	94	430	499	1590	Myocarditis
16	79	M	25	HTN, IHD, DM	Dyspnea, malaise, LOC	81%	30-40%	2	17	4.1	13.6	114	32	760	726	21	Sepsis
17	80	M	27	HTN, CRF	Fever, dyspnea, malaise, LOC	86%	40-50%	8	17	7	13.2	158	56	420	686	22	Sepsis
18	79	M	31	HTN, IHD	Fever, cough, dyspnea, malaise	77%	70-80%	4	10	5.5	14.8	115	43	496	1573	11	ARDS
19	55	M	29	DM	Cough, dyspnea	94%	30-40%	7	19	3.9	14.4	102	24	370	310	30	Sepsis
20	77	M	26	HTN, IHD	Cough, dyspnea, malaise	94%	30-40%	7	10	12.4	13.7	280	45	340	430	0	ARDS
21	75	M	24	HTN, IHD, CLD	Cough, dyspnea	80%	20-30%	7	17	6.3	12.7	190	38	248	614	21	Sepsis
22	91	M	27	IHD, CLD, CRF	Cough, dyspnea, malaise, LOC	88%	20-30%	5	17	12	9.1	288	71	1312	600	18	Sepsis
23	69	M	28	HTN, IHD, DM	Dyspnea, malaise, LOC	80%	60-70%	5	10	25	9.2	397	66	430	476	57	ARDS
24	70	M	22	HTN, IHD, DM, CRF	Cough, malaise	92%	40-50%	12	16	11.4	13.9	126	72	170	365	45	Sepsis
25	94	M	26	HTN, IHD, CRF	Cough, dyspnea	94%	20-30%	10	30	7.8	9.8	235	54	250	232	21	Sepsis
26	73	F	31	HTN, IHD, DM, CRF	Cough, dyspnea, malaise	72%	60-70%	3	15	16.3	13.4	333	73	2750	814	32	PTE
27	48	M	32	HTN, IHD, CRF	Fever, cough, dyspnea	75%	80-90%	5	12	13	13.6	178	78	890	650	41	ARDS
28	76	F	28	DM, CRF	Fever, cough, dyspnea	82%	50-60%	4	15	8.9	11.8	213	54	320	430	22	ARDS
29	78	M	21	HTN, CRF	Cough, dyspnea, LOC	73%	50-60%	7	12	14	12.7	212	54	650	280	12	ARDS
30	64	M	28	HTN, IHD, DM	Fever, cough, dyspnea	86%	50-60%	3	13	5.8	13.8	137	46	540	827	60	ARDS

CT, computed tomography; M, Male; F, Female; GGO, ground glass opacity; HTN, hypertension; IHD, ischemic heart disease; DM, diabetes mellitus; CRF, chronic renal failure; LOC, loss of consciousness; CLD, chronic lung disease.

###  Histopathological Findings

 We obtained pulmonary and cardiac tissue from each patient. Three cardiac and two pulmonary tissues failed during the sampling procedure and tissue preparations. We studied 55 tissue samples, including 28 pulmonary and 27 cardiac specimens.

###  Pulmonary Tissue

**Figure 1:**
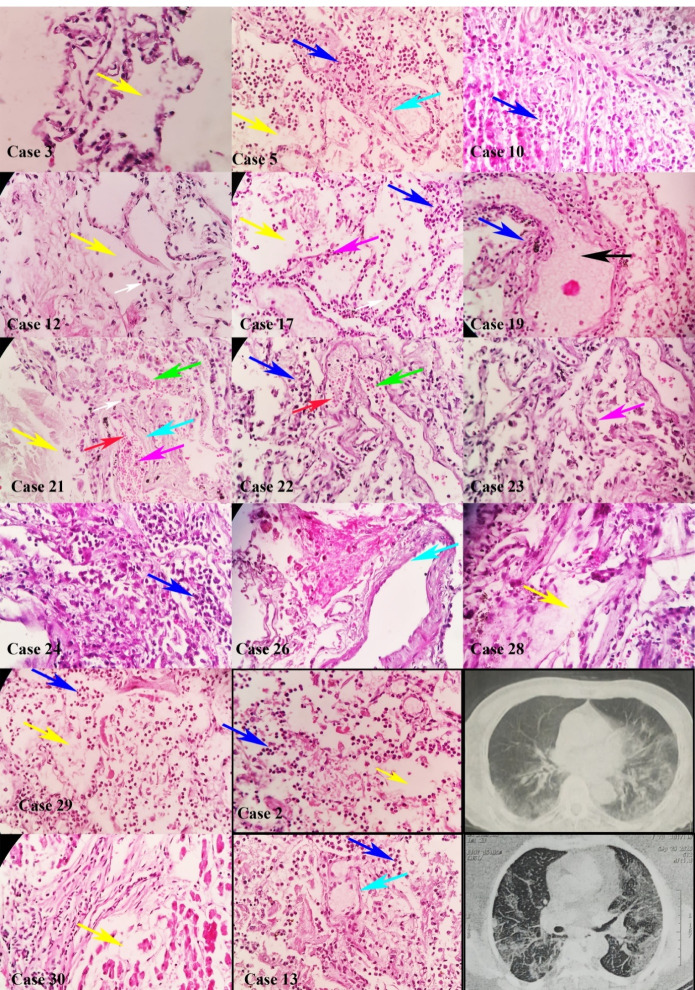
Diffuse Alveolar Damage in Pulmonary Tissues of Deceased Patients with COVID-19. Pulmonary tissues in cases 2, 3, 5, 10, 12, 13, 17, 19, 21, 22, 23, 24, 26, 28, 29, and 30 showed diffuse alveolar damage in all tissues, consisting of edema (yellow arrow), intra-alveolar hemorrhage (red arrow), neutrophil clusters (blue arrow), plasma cells (white arrow), type II pneumocyte (magenta arrow), hyaline formation (cyan arrow), and fluid accumulation (black arrow) with hematoxylin and eosin dyes. Computed tomography in cases 2 and 13 (× 100 magnification)

 We evaluated 28 samples of pulmonary tissues. Histopathological study revealed acute exudative phase in 5 (17.9%), sub-acute proliferative phase in 16 (57.1%), and late fibrotic phase in 7 patients (25%). The most common histopathologic findings in the pulmonary tissue included DAD in 28 (100%), hyaline membrane formation in 26 (92.8%), bronchopneumonia in 20 (71%), increased number of types II pneumocytes in 14 (50%), alveolar hemorrhage in 12 (42.8%), anthracosis in 11 (39.2%), and micro-thrombosis in one (4%). DAD comprised the exudative phase in 5 (17.9%), the exudative-proliferative phase in 16 (57.1%), and the fibrotic-organizing phase in 7 samples (25%) (Figure 1, Figure S2 and Figure S3).

###  Cardiac Tissue

**Figure 2:**
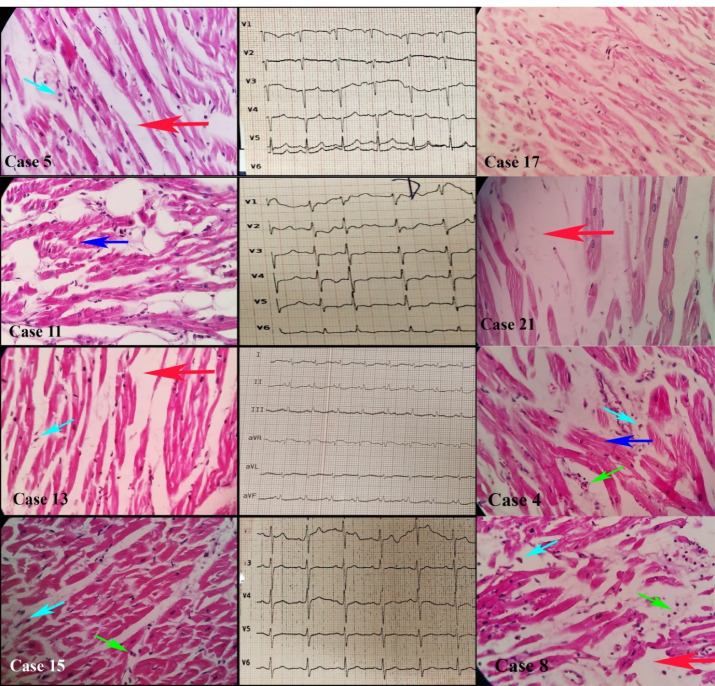
Myocarditis and Myocytes Changes in Cardiac Tissues of Deceased Patients with COVID-19. Cardiac tissues in the cases 4, 5, 8, 11, 13, 15, 17, 21 showed hypertrophic myocytes (blue arrow), interstitial edema (red arrow), myocyte necrosis (cyan arrow) in the cases 4, 5, 8, 13, and 15, and mononuclear infiltration (green arrow) in cases 4, 8, and 15, with hematoxylin and eosin dyes (× 100 magnification)

 We evaluated 27 samples of cardiac tissues. Histopathological evaluations showed normal tissue in 19 (70.3%), interstitial edema and myocyte hypertrophy in 7 (25.9%), and inflammatory cell infiltration compatible with myocarditis in 3 samples (11.1%) (Figure 2). Table 2 summarizes our patients’ main histopathological findings in pulmonary, cardiac, and cerebral tissues.

**Table 2:** Pulmonary Lesions and Cardiac Lesions

**Case**	**Pulmonary**	**Cardiac**
1	Hyaline membrane, exudative DAD, hemorrhage, appearance of pneumocytes II, anthracosis	Normal
2	Hyaline membrane, exudative and proliferative DAD, hemorrhage, appearance of pneumocytes II, bronchopneumonia	Normal
3	Exudative and proliferative DAD	Normal
4	Hyaline membrane, organizing DAD, bronchopneumonia, anthracosis	Inflammatory infiltrations, hypertrophic myocytes and necrosis
5	Hyaline membrane, exudative and proliferative DAD, bronchopneumonia	Hypertrophic myocytes, interstitial edema
6	Hyaline membrane, organizing DAD, bronchopneumonia, hemorrhage, anthracosis	Normal
7	Hyaline membrane, organizing DAD, bronchopneumonia, anthracosis	Normal
8	Hyaline membrane, exudative DAD, appearance of pneumocytes II, anthracosis	Inflammatory infiltrations, hypertrophic myocytes and necrosis
9	Hyaline membrane, exudative DAD, hemorrhage, appearance of pneumocytes II	Missing Sample
10	Hyaline membrane, exudative and proliferative DAD, hemorrhage, appearance of pneumocytes II, bronchopneumonia	Normal
11	Hyaline membrane, organizing DAD, bronchopneumonia, anthracosis	Hypertrophic myocytes, interstitial edema
12	Hyaline membrane, exudative and proliferative DAD, appearance of pneumocytes II, bronchopneumonia	Normal
13	Hyaline membrane, exudative and proliferative DAD, appearance of pneumocytes II, bronchopneumonia, anthracosis	Hypertrophic myocytes, interstitial edema
14	Hyaline membrane, exudative DAD, appearance of pneumocytes II,	Normal
15	Hyaline membrane, organizing DAD, bronchopneumonia	Inflammatory infiltrations, myocytes necrosis, hemorrhage
16	Hyaline membrane, organizing DAD, bronchopneumonia	Normal
17	Exudative and proliferative DAD, appearance of pneumocytes II, bronchopneumonia	Hypertrophic myocytes, interstitial edema
18	Missing sample	Normal
19	Hyaline membrane, exudative and proliferative DAD, hemorrhage, bronchopneumonia	Missing sample
20	Hyaline membrane, exudative DAD, appearance of pneumocytes II	Normal
21	Hyaline membrane, exudative and proliferative DAD, appearance of pneumocytes II, bronchopneumonia	Hypertrophic myocytes, interstitial edema
22	Hyaline membrane, exudative and proliferative DAD, hemorrhage, appearance of pneumocytes II, bronchopneumonia, anthracosis	Normal
23	Hyaline membrane, exudative and proliferative DAD, appearance of pneumocytes II, anthracosis	Normal
24	Hyaline membrane, exudative and proliferative DAD, hemorrhage, appearance of pneumocytes II, bronchopneumonia	Normal
25	Hyaline membrane, organizing DAD, hemorrhage, bronchopneumonia, anthracosis	Missing sample
26	Hyaline membrane, exudative and proliferative DAD, micro thrombosis, hemorrhage	Normal
27	Missing sample	Normal
28	Hyaline membrane, exudative and proliferative DAD, hemorrhage, bronchopneumonia, anthracosis	Normal
29	Hyaline membrane, exudative and proliferative DAD, hemorrhage, bronchopneumonia	Normal
30	Hyaline membrane, exudative and proliferative DAD, bronchopneumonia	Normal

DAD, diffuse alveolar damage.

 In univariate analysis by Fisher’s exact test, we did not find any association between the clinical cause of death and troponin level (*P* = 0.470), C-reactive protein (CRP) level (*P* = 0.750), GGO extension in lung CT scan (*P* = 0.392), and BMI (*P* = 0.357). Only death due to pulmonary embolism was significantly associated with a 4-fold increase in D-dimer level (*P* = 0.011). Also, we did not find any association between the DAD staging and GGO extension (*P* = 0.702), D-dimer level (*P* = 0.268), CRP level (*P* = 0.211), and BMI (*P* = 0.558). Regarding cardiac pathology, we found an association between myocarditis and a two-fold rising of troponin (*P* = 0.010) and CRP over 80 mg/dL (*P* = 0.008). However, we did not find any association between myocarditis and pulmonary GGO extension (*P* = 0.774) or BMI (*P* = 1.000).

## Discussion

 Our study is the first comprehensive multi-organ autopsy study with a generous sample size of patients with COVID-19 in Iran. Our findings revealed that all pulmonary tissues developed DAD, while 11% of the cardiac tissue samples showed inflammation. Other findings were cardiomyocyte hypertrophy with interstitial edema in the cardiac tissues. DAD occurred most often in the proliferative and organizing phases, suggesting that most patients expired during the late stage of the disease. Previous studies reported DAD in various stages, most often in the early phase of the disease. A study in Spain on 18 deceased patients with COVID-19, with a mean age of 61 years, reported that the proliferative phase of DAD occurred in 16 patients (89%), and micro-thrombosis occurred in 6 patients (33%).^9^ Another study in Austria on 14 deceased patients with a mean age of 81 years showed that the organizing phase of DAD occurred in 13 (93%), and the exudative phase occurred in 12 patients (86%). In that study, all patients developed pulmonary hemorrhage, and 11 (79%) developed bronchopneumonia and micro-thrombosis.^10^ One study in Germany on 10 deceased patients showed an acute phase of DAD in 9 (90%) and an organizing phase in one (10%).^11^ We also found anthracosis in 11 of 28 pulmonary tissues (39%). A study on eight deceased patients in the United States reported anthracosis in all samples.^12^ Other studies did not report anthracosis, and the relationship between this finding and the severity or prognosis of COVID-19 continues to be investigated.

 Earlier studies showed that numerous patients with COVID-19 have a mild elevation of D-dimer levels, but a D-dimer level higher than 2590 ng/mL was reported to be a significant risk of thromboembolism.^13^ In our study, 3 out of 30 patients (10%) had D-dimer levels higher than 2590 ng/mL, and only one of the 28 pulmonary tissues (3.5%) revealed thrombosis. The frequency of thrombosis in our pulmonary tissue samples was much lower than other reports. One study on 76 deceased patients in Hamburg showed that 32 patients (40%) developed deep vein thrombosis, and 17 patients (21%) developed pulmonary artery embolism.^14^ Another multicenter study on 68 pulmonary tissues of deceased patients with COVID-19 in the United States and Italy showed that 59 patients (87%) developed DAD, 57 patients (84%) developed micro-thrombosis, and 29 patients (43%) developed large-vessel thrombosis.^15^ A study by Carsana et al on 38 deceased patients with COVID-19 in Italy reported that all patients developed a 10-fold rise in D-dimer levels, and 33 (87%) developed thrombosis.^16^ Other studies have also reported a high prevalence of deep vein thrombosis and micro-thrombosis in deceased patients with COVID-19.^17-20^ This discrepancy may be due to differences in sampling techniques, study populations, and anticoagulant treatments. We used minimally invasive tissue sampling methods, which may underestimate the existing pathology.

 Cardiac complications are a leading cause of death in some patients with COVID-19. They may arise from exacerbation of a pre-existing cardiac disease or an acute condition during the illness, such as infarction, thrombosis or myocarditis. In our study, 7 out of 27 (25.9%) cardiac tissues showed cardiomyocyte hypertrophy, and 3 out of 27 (11.1%) samples showed myocarditis. The frequency of myocarditis in COVID-19 varies in different studies. A study on 22 cardiac samples of deceased patients with COVID-19 with a mean age of 68 years in the United States showed a mild increase of troponin in 20 patients (90.9%), but no case of myocarditis was reported.^21^ Another multicenter study in the United States and Italy reported myocarditis in 3 out of 21 patients (14.2%).^22^ A survey of nine deceased patients with a mean age of 72 years reported only one case of myocarditis (11.1%).^23^ One study on four deceased patients revealed the initial stages of myocarditis in all cardiac tissue samples.^24^

 Limited multi-organ studies were also performed on deceased patients with COVID-19. A study in South Africa on 75 deceased patients with a mean age of 60 years indicated the exudative phase of DAD in 47 (62.6%) and myocarditis in 7 (9.3%) patients.^25^ Another study on 21 deceased patients in Switzerland showed the exudative phase of DAD in 16 patients (76.2%), but no case of myocarditis was reported in cardiac tissues.^26^ A study on 11 patients in Austria showed that only one patient (9%) had myocarditis.^27^ Two additional multi-organ studies on deceased patients, including 21 deceased patients in the Netherlands and 10 deceased patients in the United Kingdom, showed myocarditis in 55% of the Dutch and 0% of the UK patients.^28,29^ Other studies on 17 and 12 deceased patients in the United States showed myocarditis in 0% and 7%, respectively.^30,31^ A multi-organ study on 22 deceased patients with a mean age of 68 years in Italy showed that 12 patients (54.5%) had myocarditis.^32^ Furthermore, a survey of 32 deceased patients with a mean age of 68 years demonstrated myocarditis in only one patient (3%).^33^ We did not find any similarity between our findings and other studies about myocarditis. In our research, myocarditis did not have a significant association with the severity of the disease. So, it may be attributed to other risk factors, such as viral tropism or race. We were unsure that all the histopathological findings were related to COVID-19, especially pulmonary anthracosis, increased type 2 pneumocytes, cardiac myocytes hypertrophy, and edema. Like the previous studies, our most common findings in the pulmonary tissues were DAD and hyaline membrane formation. The authors believe that these findings are related to COVID-19. However, other nonspecific findings such as hemorrhage, anthracosis, and the appearance of type II pneumocytes may be seen in other patients with underlying pulmonary diseases. In the cardiac tissues, the authors also believe that mononuclear infiltration in the patients with elevated cardiac troponin are related to COVID-19, as myocarditis. Nevertheless, other findings, such as myocyte hypertrophy and interstitial edema, are unspecific. So, we tried to avoid any complex analysis and only expressed our observations. Nevertheless, in contrast to previous studies, we found a lower frequency of micro-thrombosis in our pulmonary samples.

## Conclusion

 The most common pulmonary histological findings in our study was diffuse alveolar damage, similar to previous studies. Still, in cardiac tissues, we found a higher frequency of myocarditis and cardiac myocyte degeneration than in the earlier studies.

## Limitations

 The sampling technique was our major limitation Percutaneous needle biopsy provides less histopathological information compared to open biopsy and may underestimate pathological changes, such as thrombosis. Another limitation was the absence of a control group to compare our pathology findings between the COVID and non-COVID patients. Unavailability of immunohistochemical staining was also a limitation in differentiating inflammatory cells and immunological assays of the tissue samples.

## Supplementary Files

Supplementary file 1 contains Figures S1-S3.
