# Supplementary material for: Cardio-Pulmonary Histopathology with Clinical Correlations of Deceased Patients with COVID-19: A Case Series in Tehran, Iran
Source: Arch Iran Med. 2023 May 1;26(5):252–60. doi: 10.34172/aim.2023.39 (PMC10685862; doi:10.34172/aim.2023.39)
Supplement: Supplementary file 1 — contains Figures S1-S3. [file aim-26-252-s001.pdf]

Arch Iran Med. May 2023;26(5):252-260

Doi: 10.34172/aim.2023.39

# Supplementary file 1

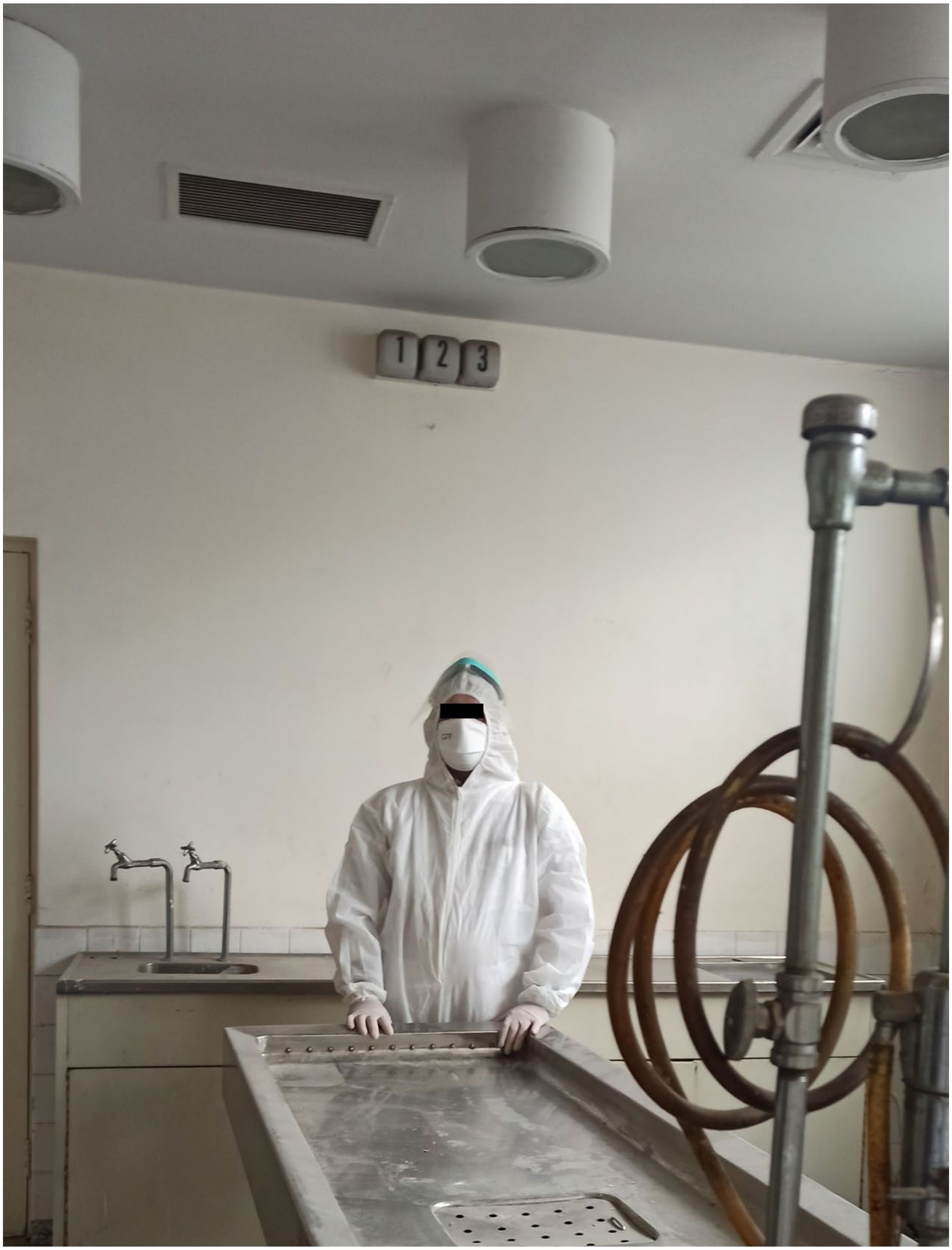


**Figure S1.** The sampling room with negative pressure ventilation.

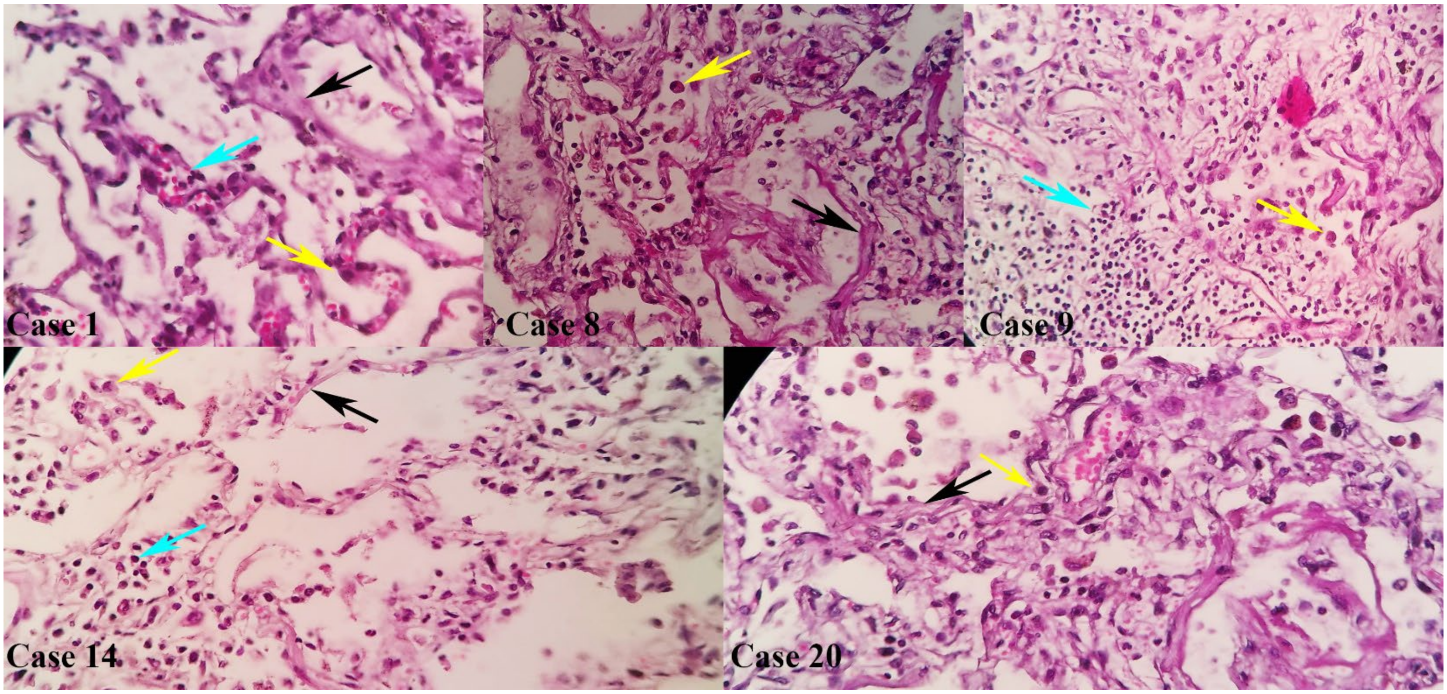


**Figure S2.** Pulmonary tissues in the cases 1, 8, 9, 14, and 20 showed exudative DAD, hyaline formation and thick alveolar wall (black arrow), Plasma cell (yellow arrow), and infiltration (cyan arrow).

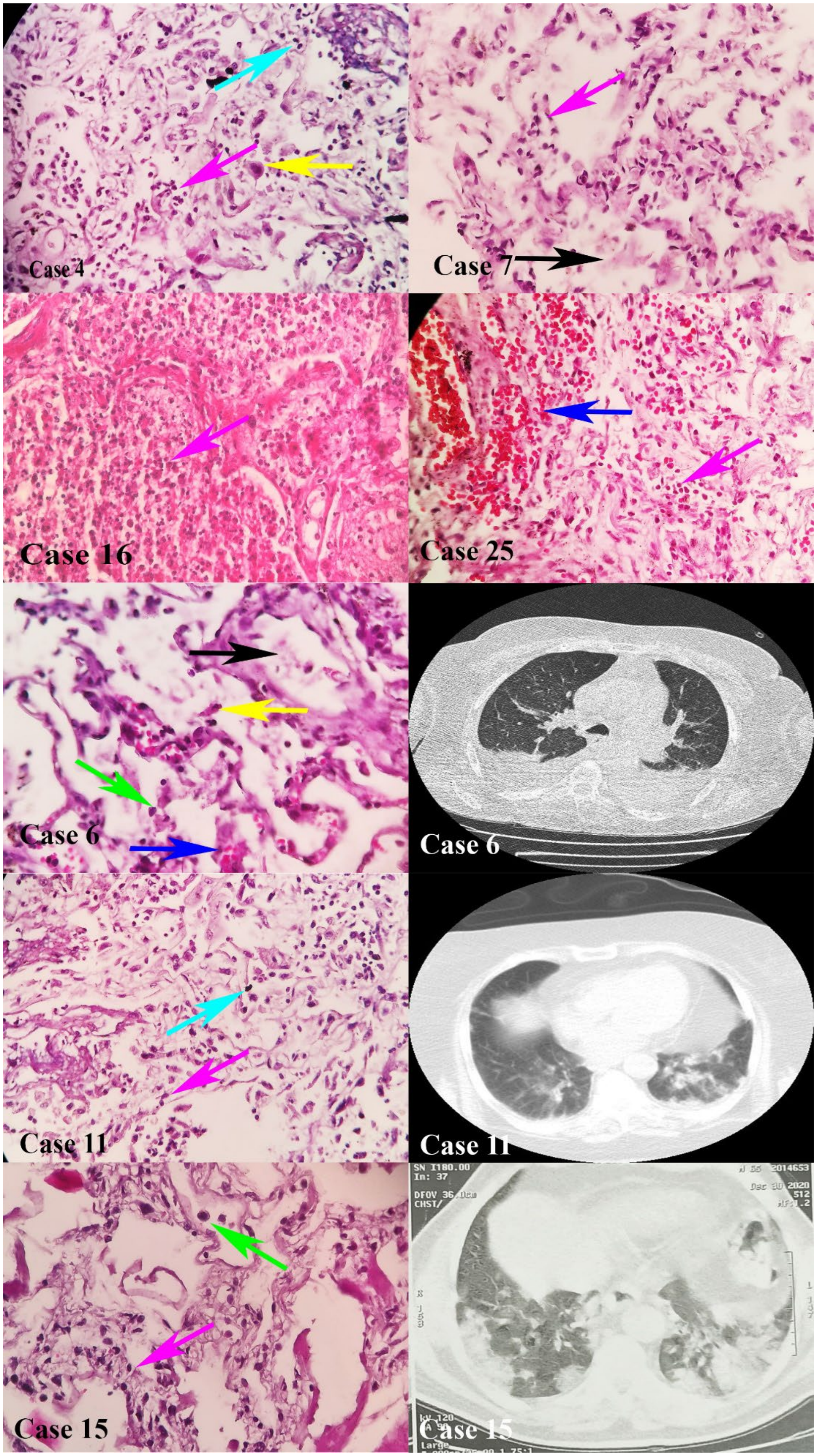
Case 4
Case 7
Case 16
Case 25
Case 6
Case 6
Case 11
Case 11
Case 15
Case 15

**Figure S3.** Pulmonary tissues in cases 4, 6, 7, 11, 15, 16, and 25 showed Diffuse alveolar damage and hyaline membrane in all tissues, fluid accumulation (black arrow), hemorrhage (blue harrow), anthracosis (cyan arrow), megakaryocytes (yellow arrow), pneumocyte type II (green arrow), and mononuclear infiltration (magenta arrow). computed tomography in cases 6, 11, and 15.
